# Quality evaluation of water disclosure of Chinese papermaking enterprises based on accelerated genetic algorithm

**DOI:** 10.1038/s41598-023-39307-y

**Published:** 2023-07-28

**Authors:** Chaoran He, Juqin Shen, Jiawei Xu, Fuhua Sun, Bing Wang

**Affiliations:** 1 Business School, Hohai University, Nanjing, 210098 China; 2 College of Agricultural Science and Engineering, Hohai University, Nanjing, 210098 China; 3 Xuchang Bureau of Hydrology and Water Resources Investigation, Xuchang, 461000 China

**Keywords:** Ecology, Environmental sciences, Environmental social sciences

## Abstract

As the carrier of enterprise water resources management disclosure, water information disclosure is a means of expression of enterprises’ environmental responsibility. First, a corporate water information disclosure quality evaluation index system and evaluation method are established, and with the help of the projection tracing method of accelerated genetic algorithm, 27 paper companies in China are selected as a sample and the disclosure quality level is analyzed empirically. Then, the analysis is carried out in terms of three changes in vertical trends, horizontal trends and changes in laws, regulations and policies, and the results show that Chinese paper and paper product enterprises have low quality of water information disclosure, weak disclosure content and low voluntary disclosure. Finally, feasible suggestions are made based on the evaluation of disclosure issues.

## Introduction

As an important traditional manufacturing industry, the paper industry is closely related to social development and ecological environment, and is also an important part of water consumption. As a disclosure carrier of corporate water resources management, water information disclosure describes the relevant information of corporate water use and drainage through monetary performance or volume, and discloses non-financial reports on corporate water management, strategy and other business risks and opportunities to stakeholders (The CEO Water Mandate, 2014), which occupies an important part of environmental information disclosure. With the intensification of global climate change and the increasing strain on industrial water use, the importance of corporate water information disclosure has made Chinese enterprises have begun to pay attention to their own water resource management, and gradually integrate water resource management into high-level supervision (Carbon Disclosure Project China, 2020). The research field of water resources management mainly focuses on the influence of macro factors on enterprise water resources management from the perspective of economics^1^, the strategy of corporate water resources from the perspective of management^2^, the improvement of enterprise water resources accounting capacity from the perspective of water management accounting^3^, and the development of comprehensive framework of enterprise water management accounting^4^. Whether in practice or theoretical research, water environment issues have gradually been paid attention to by enterprises. Green development with the goal of "water-saving priority" has become one of the important directions for the development of the paper and paper products industry.

According to the statistics compiled by the China Environmental Statistical Yearbook, the wastewater discharge of the paper industry accounted for 12.3% in 2020, second only to chemical raw materials and chemical products manufacturing, and is the second largest industry in China’s industrial wastewater discharge. Although the Administrative Measures for the Legal Disclosure of Enterprise Environmental Information reviewed by the Ministry of Ecology and Environment clarifies the accurate and complete disclosure of environmental information by enterprises. However, in the actual enterprise report disclosure, the information disclosure on water consumption, total groundwater withdrawal control and wastewater emission reduction is far lower than expected, and there are problems such as incomplete and inconsistent water information disclosure and weak water performance disclosure. Water information disclosure by enterprises not only promotes the transformation of enterprises from extensive management to refined management mode, but also optimizes the water resource management mechanism of enterprises, which is more important for sustainable water resources management^5^. With the increasing attention of society to the environment, the information disclosure of listed companies has transitioned from voluntary disclosure to mandatory disclosure, and carbon emission information must be disclosed in the content requirements of environmental information disclosure according to law, but there are few related contents related to water information disclosure, and there are no clear requirements. As a result, enterprises cannot deal with the "internal and external response" problem of water information disclosure, that is, the imbalanced ecological contradiction between enterprises and nature, and human society. In the process of papermaking production and sales activities, enterprises gradually expose the low water efficiency and slow update and iteration of water-saving technology, resulting in high production water costs, and increasing the risk of enterprises taking the social responsibility of ecological water as a marketing strategy and public relations means of "greenwashing"; It cannot meet the individualization of stakeholders and their needs, and it is not conducive to the role of the market in allocating resources^6^.

In view of the water information disclosure reports of existing enterprises, how to scientifically and objectively evaluate the quality of enterprise water information disclosure, and the disclosure of enterprise water information has become a hot spot for scholars. At present, the establishment of an applicable model of enterprise water information disclosure quality has become the most urgent need at present, this paper constructs an evaluation index system of enterprise water information disclosure by sorting out relevant literature and results at home and abroad, and at the same time, based on the quality characteristics of enterprise accounting information disclosure as a research framework. Finally, the improved projection tracing model is used to analyze the water information disclosure quality of paper and paper products enterprises and obtain the final evaluation value. This paper and paper products industry is trying to analyze whether China’s paper and paper products industry has got rid of water-consuming production methods, aiming to better summarize the current results and problems of intensive water conservation in China's paper and paper products industry.

The theoretical contribution of this paper is fourfold, first, the study is based on the high water consumption and high pollution light industry—Paper and paper products industry as an example, the introduction of sustainable development theory and environmental accounting theory and other research methods, through theoretical analysis from internal factors and external factors two perspectives a comprehensive and systematic analysis of the connotation of enterprise water information disclosure, The impact of corporate water disclosure on the evaluation of the company's own water environment and corporate value is explored to provide a theoretical basis for corporate water management and sustainable innovation; then, that is, a corporate water disclosure indicator evaluation system is constructed, which will help stakeholders to assess the understanding of optimal management of water resources within the company, bridging the gap between existing studies in the evaluation of the quality of corporate water disclosure; Next, this paper constructs a model based on projection tracing method, which can overcome the interference of subjective factors and improve the performance of computing more accurately and scientifically, and improve the algorithm as an accelerated genetic algorithm encoded with real numbers, so as to speed up the convergence speed, improve the accuracy and robustness of the model, and finally find the best projection value of the sample, rank the quality of corporate water disclosure, and solve the subsequent corporate water disclosure laying the foundation for solving the problem; Finally, this paper systematically proposed enterprise water information disclosure index system, based on the quality characteristics of enterprise environmental accounting information, from the four principles of relevance, reliability, effectiveness and comparability, to build a new paper and paper products enterprise water information disclosure Indicator system, enrich the existing literature of water information disclosure system in each level of indicators, improve the practicality of enterprise water information disclosure, and thus has a theoretical contribution to provide planning and decision-making for the sustainable and high-quality development of enterprise water resources cycle.

The rest of the paper is structured as follows: Sect. “Literature reviews” provides an overview of the factors and quality evaluation methods that affect the disclosure of corporate water information. The third section constructs the enterprise water information disclosure quality evaluation index system and model, scores based on the four principles of accounting information quality, and adopts the improved projection tracing model method. In the fourth section, the horizontal and vertical process changes of water information quality of China’s A-share papermaking and paper products enterprises are analyzed using weight indicators and final evaluation values. Finally, the research conclusions are drawn and recommendations are made for the future water management of the enterprise.

## Literature reviews

Only high-quality disclosure of water resources information can enterprises achieve long-term sustainable development. Most of the domestic and international literature focus on the current situation of corporate water information disclosure and the impact mechanism, expressed in the company’s internal and external factors. From the perspective of internal factors, corporate water disclosure is motivated by "internal incentives" and "own risk" considerations^7,8^. Internal incentives are mainly in the form of corporate governance and business performance. Liu et al.^9^ used a sample of 781 listed companies to clarify that water information disclosure through financing constraints can significantly improve financial quality, thus establish a good reputation and help enterprises develop capital markets for green credit; Japanese scholars Burritt et al.^10^ conducted an empirical study on 100 enterprises’ water information disclosure and explored the impact of corporate water risk awareness, media exposure, and ownership dispersion on corporate water disclosure behavior. Malik et al.^11^ constructed environmental information disclosure level indicators based on listed companies in China to analyze the significance of the environment on corporate performance at macro and micro levels. Liao et al.^12^, Clarkso et al.^13^ and Martinez^14^ found that corporate information disclosure was significantly and negatively related to environmental performance. It means that enterprises found to have poor business performance in the early stage should disclose environmental information promptly to avoid future penalties for not fully disclosing it. Risk is mainly manifested in the enterprise’s own risk. Zkou et al.^15^ study selected 334 listed companies in China’s high-water risk industry. The results show that water information disclosure is not significantly correlated with enterprise systemic risk, but helps enterprises to attenuate systemic risk. Most of the listed companies' business development is financed by external financing. The "green credit" policy launched by the regulatory authorities combines environmental and financial risks, raising higher standards for corporate environmental governance. It can be seen that corporate water information disclosure is more focused on water resources in terms of saving operating costs and improving short-term earnings.

From the perspective of external factors, generally manifested as direct pressure (government) and indirect pressure (media or market), driven by the results of pressure, corporate disclosure of water information is not only a requirement for stakeholders and companies to fulfill their social and environmental obligations, but also a requirement to achieve sustainable and high-quality national and corporate development. Deng Mingjun et al.^16^ analyzed the motivation mechanism of corporate water information disclosure, explained the mechanism of corporate water information disclosure regulation and realization mechanism, and called on relevant authorities to set quantifiable and enforceable evaluation criteria for critical regulations such as "significant corporate water-related impact" and "significant corporate water-related litigation". We call on relevant authorities to set quantifiable and clearly enforceable evaluation criteria for critical provisions such as "company water-related material impact" and "company water-related material litigation, Walid and Mohamed^17^. Taking a sample of 1166 non-financial companies in the CDP report, the empirical test of how national policies and legal systems can help strengthen the level of water information disclosure of enterprises, and Shen Juqin et al.^18^ constructed a water information phi level indicator system in line with China's national conditions, and verified the sensitivity of social monitoring to water information disclosure through model testing, indicating that under external environmental governance, the public and the government attach great importance to the environment and the use of information.information utilization status, and increased the willingness of enterprises to actively disclose. Xu Jianling et al.^19^ and Zeng et al.^20^, using a sample of listed companies in highly water-sensitive industries, confirmed that the effect of water information disclosure on corporate risk containment was strengthened by means of media exposure through the news. Media coverage generates reputational pressure to a certain extent, forcing firms to fulfill their environmental responsibilities. Thus, external monitoring plays a leading role in environmental governance, with the public monitoring pollutant emissions and the government compensating for market "failures" and providing subsidies and incentives in tax and credit policies, so that water conservation is elevated to the strategic level of corporate water management actions to achieve high-quality economic development and sustainable and harmonious social and environmental development.

The choice of different evaluation methods will have an impact on the accuracy of the evaluation. Scholars at home and abroad have studied the content of water disclosure through empirical research, and the relevant research results provide more options for improving the level of corporate water disclosure. And there is no unified standard for evaluating and measuring water information disclosure quality, and many scholars have explored various ways to measure the quality of corporate water information disclosure. Liu CY et al.^21^ and Yu HC^9^ took the data of Chinese listed companies as a sample, and after mathematical and statistical analysis of the disclosed water information, based on the relevant information disclosed in the annual reports of enterprises, to The "quantitative + qualitative" approach was used to analyze the factors affecting water information disclosure by listed companies based on the coding of water disclosure items by the Global Reporting Initiative (GRI). Liang et al.^22^ uses the NCA and fsQCA approach to study the joint roles of environmental regulations and other internal and external factors on the quality of environmental information disclosure. Liu Xuewen^23^ used a combination of fuzzy comprehensive evaluation and hierarchical analysis methods to construct a water environmental information disclosure index system for heavily polluting listed companies, which provided a reference for the evaluation method of enterprise water information disclosure quality, Kleinman^24^ uses Formal Concept Analysis (FCA) to study water disclosure and water stewardship in the U.S. food and beverage industry.Plumle et al.^25^ and Clarkson et al.^26^ evaluated by "content analysis method" and constructed environmental information disclosure indexes for analysis and evaluation, and the study of environmental information disclosure and quality evaluation. As we can see, domestic and foreign scholars are still exploring the methods of information disclosure quality evaluation. After the promulgation of the revised Environmental Protection Law in China, what are the main contents of water information disclosed by enterprises to the relevant departments, and what is the impact of the environmental laws and regulations, and policies introduced by the government on enterprises' disclosure of information about water resources? What is the quality of water information disclosure for high water-consuming manufacturing industries? Do manufacturing enterprises record information on water consumption, withdrawal, and consumption generated in the supply chain? The existing literature fails to explain well enough to answer the more profound questions behind corporate water information disclosure.

Given the above theoretical and practical limitations, and to implement the ecological civilization construction of intensive and sustainable use of water resources, this paper studies the paper and paper products industry, a typical high water-consuming enterprise in the manufacturing industry, digs deeper and analyzes the disclosure of water information of listed enterprises, and adopts the mainstream method of content analysis to quantify the water information of enterprises. The content of the disclosure, combined with the projection tracing model of accelerated genetic algorithm, to establish a scientific and practical quality evaluation system in line with the paper and paper products enterprises, through the evaluation of the value of high and low ranking, analysis of the quality of enterprise water information disclosure, so as to force enterprises to accelerate the development of green transformation and innovation, to provide experience and methodological guidance to achieve sustainable and high-quality development of the paper industry.

## Methods

### Evaluation index system construction

Currently, China has not yet issued a responsibility report on water information disclosure, and has been in the stage of voluntary disclosure. The enterprise water information disclosure system is not perfect. The system is not sound and generally puts the data related to water disclosure into the annual report of listed enterprises, social responsibility report, and sustainable development report, resulting in the lack of uniform standards when enterprises make the disclosure. There are differences. Based on the existing studies (Morikawa et al. and Zeng Huixiang et al.), this paper selects the four basic principles of "relevance", "reliability", "validity", and "comparability" based on the quality characteristics of corporate accounting information disclosure.

Relevance is a corporate environmental report to provide evaluation information useful to users of water resources, and companies through water environmental assets, water environmental liabilities, water environmental costs, and water environmental management impact. This water-related information to stakeholders to meet the information needs of interested parties; reliability is used to test the quality of water information disclosure, usually using third-party audit or system certification to assess this reliability is used to test the quality of water information disclosure, and third-party audits or systematic certification are often used to evaluate this information. Currently, the water information disclosure audit has not been established, so the water risk assessment, environmental violations penalties, environmental honors, patented green technology, and corporate environmental emergency plans are selected to reflect the truth and objectivity of corporate water information disclosure; validity is the daily production and operation activities of enterprises to provide information about water resources clean production status, water and environmental performance comparison, production process equipment, and staff related education and training, etc. Reflect the importance of enterprise water information disclosure, as well as enterprise transformation of water-saving equipment to show the efforts made by enterprises in energy conservation and emission reduction, to eliminate investors' doubts about whether the enterprise production process is green and environmentally friendly, the indicators to choose the enterprise water resources management performance evaluation, enterprise water conservation measures secondary indicators for effectiveness; comparability refers to different enterprises, different periods of time can be compared with each other information. Annual reports, social responsibility reports, and environmental reports as disclosure vehicles. Disclosure information will involve the degree of detailed water information disclosure, a horizontal comparison of the report's content. Content analysis method, "quantitative + qualitative" method, the number and quality of disclosure analysis, each quality dimension assigned a value range of [0 – 3], the higher the score, on behalf of the paper and paper products enterprises more detailed water information disclosure standard. The results are shown in Table 1.

**Table 1:** Enterprise water information disclosure quality index system.

Guideline indicators	Tier 1 indicators	Secondary indicators	Evaluation criteria
Relevance	Water environment assets (A1)	Water source (B1)	For the area where the water source is sufficient, take the value of 3, generally take the value of 2. For local water shortage 1, water scarcity takes the value of 0
		Water recycling efficiency (B2)	Quantitative and qualitative disclosure of water pollution control and emission reduction information to take the value of 3, quantitative hit 2, qualitative hit 1, otherwise take the value of 0
		Environmental investment (B3)	Quantitative and qualitative disclosure of water pollution control and emission reduction information to take the value of 3, quantitative hit 2, qualitative hit 1, otherwise take the value of 0
	Water environment liabilities (A2)	Water resources tax or environmental protection tax (B4)	Disclosure of the amount of taxes paid quantitative and qualitative disclosure of water pollution control, and emission reduction information is taken as quantitative hit 2, qualitative hit 1, otherwise the value of 0
	Water environment costs (A3)	Wastewater abatement treatment expenditure (B5)	Quantitative and qualitative disclosure of water pollution control and emission reduction information to take the value of 3, quantitative hit 2, qualitative hit 1, otherwise take the value of 0
		Enterprise water consumption (B6)	Quantitative and qualitative disclosure of water consumption row information to take the value of 3, quantitative hit 2, qualitative hit 1; otherwise, take the value of 0
		Enterprise wastewater discharge (B7)	Quantitative and qualitative disclosure of wastewater discharge information takes the value of 3, quantitative hit 2, qualitative hit 1; otherwise, it takes the value of 0
		Enterprise COD emissions (B8)	Quantitative and qualitative disclosure of COD information takes the value of 3, quantitative hit 2, qualitative hit 1, otherwise it takes the value of 0
	Water environment management (A4)	Enterprise daily water management system plans, goals and strategies (B9)	Disclosure of the company to develop water resources objectives and the completion of the system and other quantitative and qualitative disclosure of water pollution control and emission reduction information to take the value of 3, quantitative hit 2, qualitative hit 1, otherwise take the value of 0
		Environmental information exchange with stakeholders (B10)	Disclosure of environmental information exchange with environmental protection departments at all levels, suppliers, vendors, the public, industry peers, and employees quantitative and qualitative disclosure of water pollution control and emission reduction information take the value of 3, qualitative hit 2, quantitative hit 1, otherwise, take the value of 0
Reliability	Water environment regulation (A5)	Water risk assessment (B11)	Searching Baidu library and news reports, information such as positive news reports on the existence of physical risk, regulatory risk, and reputational risk of enterprises take the value of 1, negative reports otherwise 0
		Penalties for environmental violations (B12)	Disclosure of company environmental penalty information takes the value of 1; otherwise, it is 0
		Environmental honors (B13)	Get environmental protection and water conservation honor to take the value of 2, general environmental protection honor for 1, otherwise take the value of 0
		Green patented technology (B14)	The value of the green patent is 1; otherwise, it is 0
		Enterprise environmental emergency plan (B15)	Disclosure of information on the company's emergency mechanism, emergency measures, and programs for major environmental emergencies takes a value of 3 in detail, 2 for quantitative, and 1 for qualitative. Otherwise, it takes the value of 0
Validity	Water environment performance (A6)	Enterprise water management performance evaluation (B16)	Qualitative and quantitative disclosure of information on the status of clean production of water resources, comparative performance of the water environment, production processes and equipment, as well as staff-related education and training is valued at 3, quantitative hit 2, qualitative hit 1; otherwise, it is valued at 0
		Enterprise water conservation measures program (B17)	Qualitative and quantitative disclosure of detailed water conservation measures take the value of 3, quantitative hit 2, qualitative hit 1; otherwise, take the value of 0
Comparability	Disclosure vehicle (A7)	Corporate annual report (B18)	Qualitative and quantitative disclosure of relevant water information to take the value of 3, quantitative hit 2, qualitative hit 1; otherwise, take the value of 0
		Social responsibility report (B19)	Qualitative and quantitative disclosure of relevant water information to take the value of 3, quantitative hit 2, qualitative hit 1; otherwise, take the value of 0
		Environmental report (B20)	Qualitative and quantitative disclosure of relevant water information to take the value of 3, quantitative hit 2, qualitative hit 1; otherwise, take the value of 0

Among the secondary indicators of enterprise water information disclosure quality, we selected water recycling efficiency, enterprise COD emissions, environmental information exchange with stakeholders, enterprise daily water management system plans, goals and strategies, and enterprise water management performance evaluation programs as key indicators for evaluation, which reflect the key aspects of enterprise water use, water conservation measures, water discharge and wastewater treatment performance. By focusing on these key indicators, companies can better understand their core water management issues and take targeted improvement measures.

(1) Water recycling efficiency can promote the sustainable use of water resources. By improving water recycling, enterprises can reduce the over-exploitation of natural water bodies and save production costs. By optimizing the recycling technology of water resources, enterprises can demonstrate their innovative ability in resource management and competitive advantages in sustainable development, and enhance their brand image and market competitiveness. (2) COD emissions from enterprises is an important indicator for internationally common detection of the content of organic matter in water bodies and the pollution level of water quality. Monitoring COD emissions helps enterprises identify potential water environmental risks, timely identify the source of water pollution, adjust treatment plans and ensure the safety of discharged water quality. By reducing COD emissions, enterprises can reduce wastewater generation and treatment costs, improve resource utilization efficiency, and achieve the dual benefits of energy saving and cost saving. (3) Communicating environmental information with stakeholders is a social responsibility that enterprises should undertake, reflecting their concern for environmental protection and sustainable development. On the one hand, it can improve the transparency of information disclosure and convey to stakeholders the performance and responsibility of the enterprise in water resources management, which helps to enhance the reputation of the enterprise. On the other hand, by communicating environmental information disclosure with stakeholders and introducing their opinions and suggestions, it increases the diversity and legitimacy of decision making to help enterprises better respond to the expectations of society. (4) An enterprise's daily water management system plan, objectives and strategies can clarify the enterprise's goals and directions in water resources management and provide guidance for the development of specific action plans and strategies in water resources management. At the same time, it provides a set of systematic strategies and measures to help companies manage and use water resources effectively. In addition, by developing a daily water management system plan, objectives and strategies, companies are better able to identify and manage the risks associated with water management. This helps companies comply with relevant laws and regulations and reduce the legal risks they face due to non-compliance with discharge or excessive discharge of pollutants from water bodies. (5) Enterprise water management performance evaluation provides a quantitative assessment of the enterprise's internal water management practices. Through information on the status of clean production of water resources, comparative water and environmental performance, production processes and equipment, and employee education and training related to water conservation, companies can identify problems and opportunities for improvement, which can help them understand the effectiveness and efficiency of their water management and promote continuous improvement and optimization of water management strategies. The results are shown in Table 1.

Corporate water information data from the annual reports, social responsibility reports, and sustainability reports of listed companies in Juchao Consulting and Hexun.com, green patent data from China "CSMAR Solution V4.4", water risk assessment based on the Annual Report and Baidu News Database.

### Model specification

The projection tracing model was first proposed and conducted by U.S. professors Kruskal^27,28^ in the 1970s as an operational model for cluster analysis to solve the problem of high-dimensional data, many of which do not satisfy the assumption of normality and need to be solved by a robust or non-parametric method. It mainly involves projecting high-dimensional data onto a low-dimensional subspace by some combination, using a projection indicator function to describe the probability size of a certain classification ranking structure of the original system, finding the projection value that makes the projection indicator function optimal, and then analyzing the characteristics of the classification structure of high-dimensional data according to that projection value^29^. This method is used in water quality evaluation, land resource carrying capacity, and enterprise accounting information quality evaluation. In this paper, a projection tracing model based on an accelerated genetic algorithm is used to evaluate the quality of corporate water information disclosure. The specific process of model construction is as follows^30^.

Step 1: Normalization of sample indicators. Let the sample set of each indicator value be $\{ x*(i,j)|i = 1,2, \cdots ,n;j = 1,2, \cdots ,p\}$, Among them $x*(i,j)$ is the value of the Jth indicator for the ith sample, N is the number of samples, and P is the number of indicators in the sample. For the larger and better indicators, the normalization criteria were applied to the indicators using Eq. (1).

1 $$x(i,j) = \frac{{x*(i,j) - x_{\min } (j)}}{{x_{\max } (j) - x_{\min } (j)}},$$

which $x_{\min } (j)$, $x_{\max } (j)$ are the minimum and maximum values of the Jth index value in the sample set, respectively, $x(i,j)$ is the sequence of indicator eigenvalues normalized.

Step 2: Construct the projection indicator function. The projection tracing method is to project the P-dimensional data $\{ x(i,j)|j = 1,2, \cdots ,p\}$ into the low-dimensional space, Integrated into a one-dimensional projection with $a = (a\left( 1 \right),a\left( 2 \right), \ldots ,a(p))$ as the projection direction $z(i)$.

2 $$z(i) = \sum\limits_{j = 1}^{p} {a(j)x(i,j)} ,i = 1,2, \cdots ,n$$

Then the one-dimensional scatterplot is classified according to Eq. (2) as a unit length vector for the projection function expressed as

3 $$Q(a) = S_{z} D_{z} ,$$

4 $$S_{z} = \sqrt {\frac{{\sum\limits_{i = 1}^{n} {(z(i) - Ez)^{2} } }}{n - 1}} ,$$

5 $$D_{z} = \sum\limits_{i = 1}^{n} {\sum\limits_{j = 1}^{n} {(R - r_{ij} )} } u(R - r_{ij} ),$$

where $S_{z}$ is the standard deviation of the projection $z(i)$,$D_{z}$ is the local density of the projection $z(i)$; $Ez$ is the mean of the projection, $R$ is the window radius of the local scatter density. $r_{ij}$ denotes the distance between samples, $r_{ij} = |Z_{i} - Z_{j} |$,$u(R - r_{ij} )$ is a first-order unit step function, When $R - r_{ij} \ge 0$, Its value is 1; $R - r_{ij} < 0$, Its value is 0.

Step 3: Optimize the projection direction. After determining the sample index value, the $Q(a)$ maximum direction is estimated as the best projection direction,

6 $$\max Q(a) = s(z) \cdot d(z),$$

7 $${\text{s.t.}}\sum\limits_{j = 1}^{p} {a^{2} (j) = 1} ,$$

Step 4: Optimal alignment of samples. According to the optimal projection direction, the projection eigenvalue $z(i)$ of each evaluation index combined is calculated.

The genetic algorithm introduces the simulated biological evolution process into the middle algorithm, which is first converted into genetic space. After composing individuals or chromosomes according to the structure of biological evolution, the computational process is optimized by binary coding without restricting the objective function and constraints. Compared with traditional and projection tracking models, its advantages are (1) accelerated genetic algorithms are able to search and improve solutions in the space of feasible solutions, thus improving the multi-dimensional spatial movement tendency of feasible solutions, exploring diverse solutions, and discovering high-quality projections, which can improve the accuracy and robustness of the model, (2) avoiding the drawbacks of traditional projection tracking models that fall into local extremes and early global convergence. It has inherent parallelism and global superiority seeking capability. (3) Accelerated genetic algorithms are able to speed up convergence in each generation iteration and improve the efficiency of finding optimal projections by using genetic operations such as crossover and mutation to complete the optimization process in a limited amount of time. In this paper, an accelerated genetic algorithm is used to solve complex nonlinear optimization, improve the algorithm's performance in finding the best, and solve the high-dimensional global optimum problem. The basic procedure is as follows^31,32^.

First, the encoding of model real numbers is constructed, and linear optimization is performed:

$x(j) = a(j) + y(j)(b(j) - a(j)),(j = 1,2...,p)$; Then, the initial parent basic group is produced, and N initial string structure data are randomly generated.

$$\{ x*(i,j)|i = 1,2, \cdots ,n;j = 1,2, \cdots ,p\}$$, A further population of N individuals is generated for evolutionary sorting $$\{ f(i)|i = 1,2, \cdots ,n;\}$$$$\{ y(i,j)|i = 1,2, \cdots ,n;j = 1,2, \cdots ,p\}$$;

Assessment of the degree of adaptation. The merits and demerits of the individual or solution.

$F(i) = 1/(f^{2} (i) + 0.001)$; and select the next generation of individuals.

After selecting good individuals from each chromosome, the cycle was repeated—forming a new individual $P_{S} (i) = F(i)/\sum\nolimits_{{i = 1}}^{n} {F(i)} .$

By cross-operation, $Y_{2} (i,j) = u_{1} y(i_{1} ,j) + (1 - u_{1} )y(i_{2} ,j) < 0.5$

$$Y_{2} (i,j) = u_{2} y(i_{1} ,j) + (1 - u_{2} )y(i_{2} ,j),u_{3} \ge 0.5.$$

Mutation operation $$Y_{3} (i,j) = u(j),u_{m} < p_{m} (i)$$;$$Y_{3} (i,j) = u(j),u_{m} \ge p_{m} (i)$$;

Finally iterate. The cycle is repeated until the best individual in the group of N individuals is found and the operation is finished.

## Results and discussions

### Vertical changes in water disclosure for paper and paper product companies

**Figure 1:**
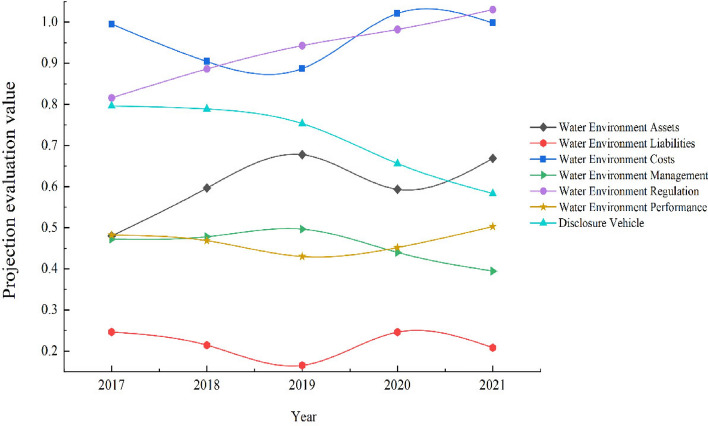
Dynamic evolution of the quality of water disclosure by paper and paper product companies, 2017–2021.

Using 2017–2021 (excluding ST, ST*, and current year listed companies) as sample data, a projection tracing model with an accelerated genetic algorithm was used to analyze the change in the trend of information disclosure about water resources in listed companies of paper and paper products. MATLAB 2022 was used to program the data, and the parents' initial population size was 200, the crossover probability was 0. 8, the variation probability was 0.1, the number of outstanding individuals was selected as 20, and the number of accelerations was 20. Obtain the best projection direction of the indicator $a^{*}$ = (0.1157, 0.2559, 0.1702, 0.1985, 0.2042, 0.3147, 0.2496, 0.3384, 0.1782, 0.2317, 0.1436, 0.1964, 0.1240, 0.2079, 0.1993, 0.2215, 0.2676, 0.2359, 0.2617, 0.2178), and to obtain the value of the investment evaluation of each sample corresponding to the disclosure of water resources information (Fig. 1).

### Changes in the vertical projection value of water information disclosure of paper and paper product companies

From the characteristics of the projection value change trend of water information disclosure of paper and paper product enterprises over the years, the projection value of each subsystem shows a staggered state of increase and decrease. Among them, the change trend of the disclosure carrier projection value shows a gradual decrease, as shown in Fig. 1. Water environment regulation, water environment assets, water environment management projection value in 2017 to 2019 overall steadily increasing, especially the water environment assets projection value from 0.4802 to 0.6778, 2019–2021, except for the disclosure carrier and water environment management projection value, showing a slow decline after a continuous increase, mainly because of the " COVID-19", in order to reduce the spread of the virus, some localities implemented blockade and quarantine measures, which restricted the daily operations of enterprises, and as the epidemic was controlled, the Chinese government enacted policy measures to stimulate economic growth in order to promote economic recovery and development. As a result, various projection values gradually continued to rise during this period. The disclosure carrier and water environment management showed a gradual decline in the projection value of enterprise water information disclosure, decreasing from 0.7963 and 0.4724 in 2017 to 0.5833 and 0.3946, respectively. Although in 2020, the government issued the Opinions on Further Improving the Quality of Listed Companies (hereinafter referred to as "Opinions") and the Measures for the Administration of the Legal Disclosure of Enterprise Environmental Information, which put forward the principle of "the disclosure of information on listed companies". The government issued the "Opinions on Further Improving the Quality of Listed Companies" (hereinafter referred to as "Opinions") and the "Measures for the Administration of Legal Disclosure of Corporate Environmental Information" in 2020, which put forward the requirement of "improving the quality of information disclosure" to further optimize and regulate the activities of legal disclosure of corporate environmental information. However, the disclosure content does not clearly regulate the disclosure of enterprise water information, focusing more on the ecological and environmental information of the production process and carbon emission information of enterprises. Because it is not mandatory for enterprises to disclose information on water consumption, sewage discharge, etc., resulting in paper companies to disclose less content about water resources in the relevant reports, so the disclosure carrier and water environment management projection value continued to decrease from 2017 to 2021, indicating that there are certain risks in the process of using water resources.

From the dynamic evolutionary change characteristics of the projection value, the following five influencing factors are mainly summarized (1) From the analysis of tax policy factors, the Chinese government has implemented a series of tax reduction and fee reduction policies for paper enterprises, aiming to help enterprises develop green, promote transformation and upgrading and environmental protection. For the purchase and installation of qualified wastewater treatment equipment, they can enjoy the VAT reduction policy, and the expenditure related to the purchase and installation of wastewater treatment equipment by enterprises can be included in the cost or offset against income tax. In addition, the Chinese tax law stipulates that enterprises purchasing fixed assets (such as wastewater treatment equipment) can charge depreciation expenses according to certain depreciable life. The depreciation expense can reduce the tax burden of enterprises and promote the return of investment. While enjoying tax benefits, in order to restrain the ecological and environmental problems caused by the discharge of enterprise pollutants, the Chinese government started the environmental protection tax on January 1, 2018, to force environmental polluters to reduce pollution and prompt enterprises to transform and upgrade through the imposition of environmental protection tax; (2) from the analysis of technological innovation and application factors, Chinese paper enterprises adopt more efficient and environmentally friendly pulp production technologies, paper-making equipment and processes in the production process. To improve the efficiency, quality and paper manufacturing efficiency, quality and diversity of pulp production. This reduces environmental pollution and resource consumption. (3) From the analysis of industrial structure transformation factors, Chinese paper companies gradually promote the digital and intelligent transformation of paper-making process equipment and apply technologies such as information technology, big data analysis and artificial intelligence in order to move toward high-quality development of comprehensive strength. Through the implementation of intelligent manufacturing and water supply chain management, they have improved the efficiency of water recycling and daily water management, and explored a new path to develop a circular economy; (4) from the analysis of environmental governance investment factors, as social awareness of environmental protection increases, more and more enterprises are aware of the importance of environmental governance and take on environmental responsibility. Some paper enterprises have a certain awareness of environmental governance and corporate social responsibility, and voluntarily increase the investment in environmental governance funds. For example, Jingxing Paper and Rongsheng Environmental, according to the annual report information statistics 2017–2019, the amount of investment in environmental protection increased from 66.35 million yuan to 127.3296 million yuan, the investment in environmental governance funds can not only promote water-saving technology innovation and improvement, improve the efficiency and effectiveness of water environmental management, but also reduce resource waste and pollution emissions, reduce costs and increase efficiency, and increase the competitive advantage of enterprises. Among other things, banks and financial institutions provide certain support to environment-friendly enterprises, including the provision of green credit and financing channels. This injects "living water" into the cash flow of paper enterprises and supports them to carry out environmental management. (5) In terms of public participation, Chinese paper companies have established a series of public participation mechanisms for external corporate management, such as public hearings, media coverage and third-party assessments. These mechanisms provide channels and platforms for public participation, enabling the public to directly participate in the environmental decision-making and management of the company and enhancing public participation. In terms of internal management, the company organizes and holds employee water conservation education and publicity activities, community involvement activities and public welfare projects to promote public attention and participation in the paper-making enterprise.

### Changes in horizontal projection values of water information disclosure of paper and paper product companies

Considering that 2020 is the closing year of China's 13th Five-Year Plan, the paper and products enterprises in 2020 are taken as the research unit, following the above approach, Exploring the characteristics of corporate water disclosure in 2020, obtaining the best projection direction of the indicator $a^{*}$ = (0.2195, 0.2680, 0.1435, 0.1894, 0.2933, 0.2490, 0.1891, 0.2856, 0.2298, 0.1802, 0.2925, 0.2066, 0.2313, 0.2621, 0.1265, 0.1465, 0.2870, 0.1631, 0.1314, 0.2424). In turn, the distribution ratio of each index dimension of the paper company is obtained.

**Figure 2:**
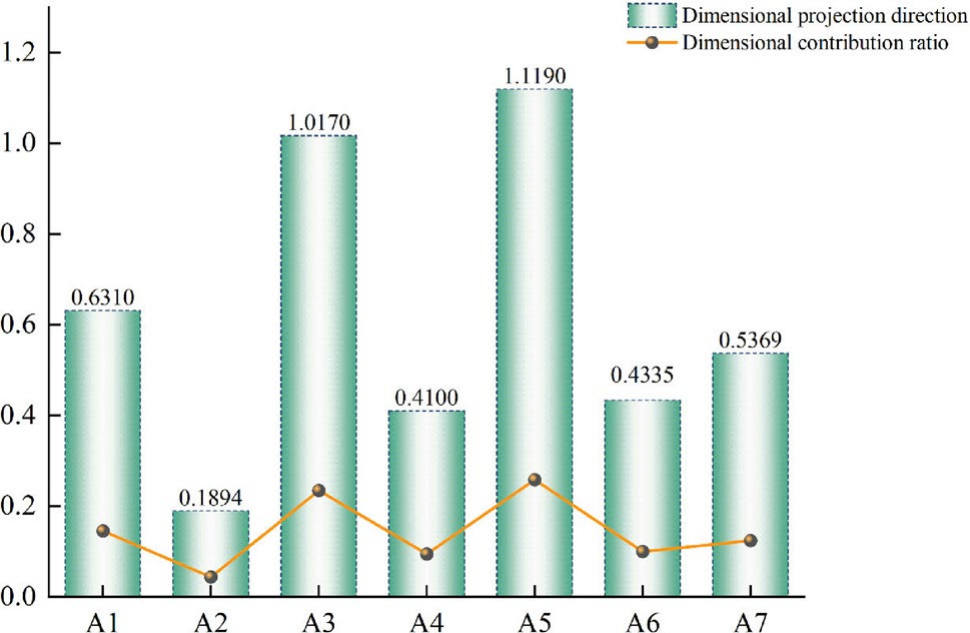
Projection weights of first-level evaluation indicators.

The value of each vector in the best projection direction represents each indicator's corresponding weight, and each indicator’s importance in evaluating the quality of corporate water disclosure can be derived by ranking. As can be seen from Fig. 2, the weight of the first-level evaluation indicators is ranked from largest to smallest as water environmental supervision (A5), water environmental costs (A3), water environmental assets (A1), disclosure vehicle (A7), water environmental performance (A6), water environmental management (A4), water environmental liabilities (A2), of which the total contribution of the three indicator dimensions of water environmental supervision, water environmental costs, and water environmental assets is more than 50%. It can be seen that listed companies in the paper industry should improve the information disclosure quality of these three indicators, which have a greater impact on the overall quality of corporate water information disclosure. The greater the indicator's weight represents a greater impact on the quality of corporate water disclosure, specifically in the secondary indicators. Table 2 shows the degree of impact of each indicator in detail. Four indicators, such as wastewater abatement treatment, water risk assessment, enterprise COD emissions, and enterprise water conservation measures program, have higher weight values and have the greatest impact on the quality of water information disclosure of paper enterprises. In addition, it can also be more intuitive to see the water information disclosure quality evaluation value projection value of paper-listed enterprises, as shown in Table 3. In order to qualitatively evaluate the quality of water disclosure of enterprises, concerning relevant literature studies^33^, this study uses a 4-level grading scale to evaluate (Table 4), and the projection values are divided into intervals, (~ , 1.6], [1.6, 2.4], [2.4, 3.2], [3.2, ~) indicating poor, average, better, and excellent.

**Table 2:** Optimal projection directions for the secondary evaluation indicators of water information disclosure for paper companies in 2020.

Evaluation indicators	Best projection direction	Evaluation indicators	Best projection direction	Evaluation indicators	Best projection direction	Evaluation indicators	Best projection direction
B1	0.2195	B6	0.2490	B11	0.2925	B16	0.1465
B2	0.2680	B7	0.1891	B12	0.2066	B17	0.2870
B3	0.1435	B8	0.2856	B13	0.2313	B18	0.1631
B4	0.1894	B9	0.2298	B14	0.2621	B19	0.1314
B5	0.2933	B10	0.1802	B15	0.1265	B20	0.2424

**Table 3:** Evaluation value of water information disclosure quality of paper companies.

Code	Company name	Rate the value	Ranking	Code	Company name	Rate the value	Ranking
002067	Jingxing Paper	3.7901	1	002228	Hexing Packaging Printing	1.2537	16
600235	Minfeng Special Paper	3.7072	2	600963	Yueyang Forest & Paper	1.2382	17
603165	Rongsheng Environmental Protection Paper	2.9978	3	002303	Mys Environmental Protection & Technology	1.2355	18
002078	Sun Paper	2.4780	4	600793	Yibin Paper	1.2311	19
000488	Chenming Paper	2.4610	5	002831	YUTO Packaging Technology	1.2181	20
002012	Kan Specialities Material	2.4550	6	002521	Qifeng New Material	1.2167	21
600433	Guanhao High-Tech	2.2351	7	603863	Songyang Recycle Resources	0.7507	22
600308	Huatai Paper	2.1004	8	002799	Global Printing	0.6766	23
600966	Bohui Paper	1.6854	9	603687	Great Shengda Packing	0.6217	24
600567	Shanying intl	1.6685	10	603022	Xintonglian Packaging	0.5863	25
002511	C&s Paper	1.6631	11	002565	Shunho New Materials Technology	0.5541	26
600103	Qingshan Paper	1.6581	12	603607	Jinghua Laser Technology	0.5355	27
600356	Hengfeng Paper	1.6333	13	–	–	–	–
000815	MCC Meili Paper	1.2662	14	–	–	–	–
603733	Xianhe Limited Ltd	1.2556	15	–	–	–	–

**Table 4:** Quality rating of water information disclosure of paper companies.

c	Grade	Number of companies	Percentage of
< 1.6	Bad	14	51.85%
1.6 ~ 2.4	General	7	25.93%
2.4 ~ 3.2	better	4	14.81%
> 3.2	Excellent	2	7.41%

From the evaluation values (see Table 3), we can see that the top three companies are "Jingxing Paper", "Minfeng Special Paper" and " Rong sheng Environmental Protection Paper", with evaluation values of 3.7901, 3.7072, and 2.9978, respectively. In the original information, these three companies issued not only independent social responsibility reports, but also disclosed independent annual environmental reports, describing in detail the content of environmental accounting, including investment in clean production, governance costs, environmental research and development expenditure and investment in environmental education and training; water resources management performance. Including water consumption, sewage discharge methods, whether the number of emissions and emission concentrations comply with national standards, water conservation program measures, water environment management, including internal environmental management system, system and clean production status, environmental protection supervision letter work, and other content. Among them, Jing xing Paper publicly disclosed in the 2020 environmental report to exchange water environmental information with stakeholders, effectively allowing public participation in the enterprise's water environmental protection work, timely follow-up and feedback on problems, and the establishment of a set of internal water environmental management system in line with the enterprise, and got 95% of stakeholders in 2020 to evaluate Jingxing Pape’s environmental information good. It indicates that the enterprise has a strong voluntary disclosure of water information and values the direct impact of water management on its sustainable and high-quality development.

From the grade rating (see Table 4), the maximum value of 27 paper enterprises is 3.7901. The minimum value is 0.5355. The average value is 1.6360, the variance is 0.804, and the standard deviation is 0.8966, indicating that the quality of water information disclosure of paper enterprises varies greatly. The difference is a bifurcated phenomenon. Only two enterprises (7.41%) that reach the excellent grade, better and general enterprises, have 11, respectively, accounting for 14.81% and 25.93%. In contrast, the majority of enterprises are bad grades, a total of 14, accounting for half of the overall proportion of 51.85%. It can be seen that most paper enterprises are missing water information disclosure content, facing low-quality of water information disclosure, and the need to improve the enthusiasm for enterprise water information disclosure.

**Figure 3:**
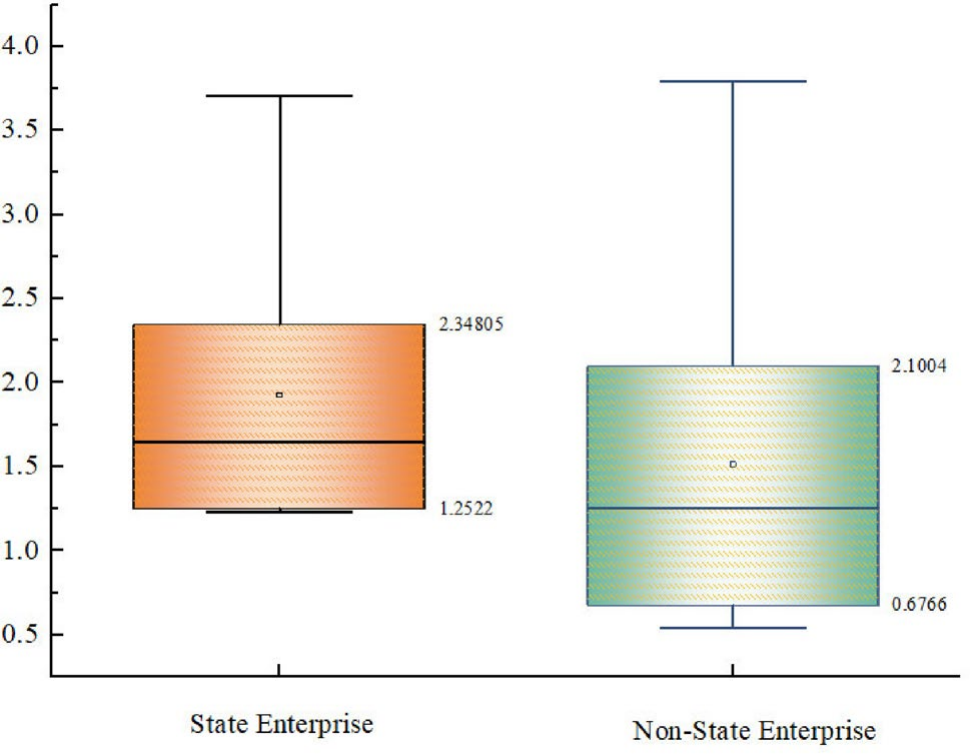
Evaluation of the nature of the enterprise of the quality of water information disclosure of paper companies.

In terms of the nature of enterprises (see Fig. 3), although the maximum projection value of SOEs is smaller than the maximum projection value of non-SOEs, the overall disclosure of SOEs is slightly higher than that of non-SOEs. Minfeng Special Paper proposes an environmental management grid in the disclosure information, establishes a special environmental management committee, incorporates the results of monthly supervision and inspection into the monthly performance assessment, and the disclosure information also makes special explanations about the situation of letters and visits during the period of Central Ecological and Environmental Protection Inspectors. In addition, Guanhao High-Tech has prepared annual sustainability reports in accordance with the Global Sustainability Reporting Standards (GRI Standards) of the Global Sustainability Standards Board, and implement the concept of efficient use of water resources and strict protection of the environment, relatively speaking, SOEs are subject to more stringent regulation and supervision in their operations. The government, as the major shareholder of SOEs, usually requires more information disclosure from SOEs to ensure a fair, just and transparent market environment. At the same time, SOEs are more sensitive to the government’s environmental policies and have better execution, especially those that have been listed for a longer period of time, pay more attention to fulfilling their social responsibility, achieving high-quality development, and carrying out environmental protection and ecological sustainability, and their image and reputation have a direct impact on the country’s image.

**Figure 4:**
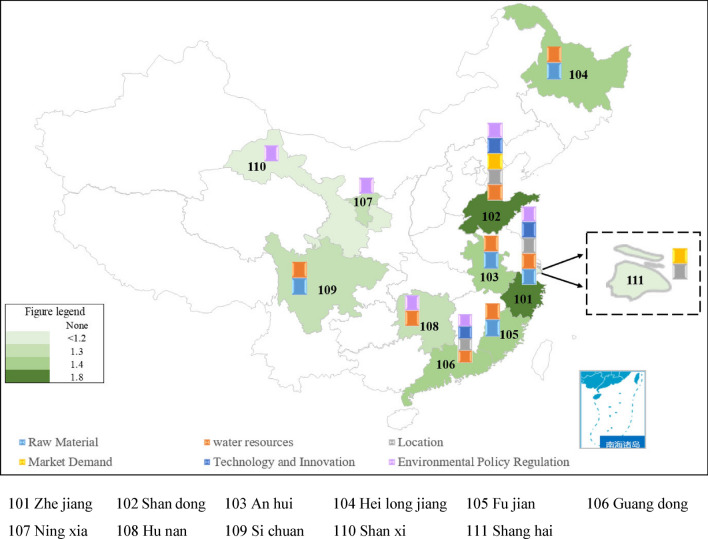
Regional evaluation of paper companies’ water information disclosure quality enterprises.

Regionally (see Fig. 4), A-share listed paper and paper products companies are concentrated in 11 provinces (municipalities and autonomous regions), with Zhejiang Province and Shandong Province generally having a higher composite rating value of 2.1947 and 1.9883 than other regions. Shanghai, as China's economic and financial center, has a dominant position in industry. However, Shanghai's water resources are mainly dependent on external water diversions and water management may focus more on meeting industrial and urban water supply needs, with relatively little attention to water information disclosure, which, combined with the fact that some water information may involve commercially sensitive information or privacy issues, may limit the scope and content of water information disclosure, resulting in a low overall rating value. From the perspective of raw materials, Heilongjiang has abundant forest resources, while Fujian and Zhejiang have abundant bamboo resources, and Guangdong and Zhejiang also have abundant waste paper resources. The abundance of these raw material resources provides convenience and cost advantages for local paper enterprises; from the perspective of water sources, Ningxia and Shaanxi land belongs to the structural water shortage, and Shanghai, although located in the coastal area, belongs to the supply and demand water shortage. In addition to these three places, the rest of the regions are abundant in water resources, which makes it convenient for enterprises to use water locally and also meet the production water needs of industrial enterprises; from the perspective of geographical advantageous location, Guangdong, Zhejiang, Shandong and Shanghai are located in the coastal areas of China, which makes it easy to trade with domestic and foreign markets. Provides convenient conditions for logistics and sales of paper enterprises; from the perspective of market demand, Shandong and Shanghai areas, the demand for packaging paper, printing paper, etc. is large, which provides local paper enterprises with broad market opportunities and potential. From the perspective of technological innovation, Zhejiang Province has strong technical strength and innovation capability in the field of specialty paper and printing paper, Guangdong Province has strong technical advantages in the field of paper processing and packaging, Shandong Province is the first in pulp and paper production nationwide, and its regional enterprises jointly complete the "high-performance wood chemical pulp green preparation and high-value utilization of key technologies and industrialization "project won the 2020 National Science and Technology Progress Second Prize, the only award-winning project in the paper industry; from the perspective of environmental policy regulation, six provincial governments have long supported and encouraged the paper industry, promoting industrial development through policy, financial and tax support. These provinces may enjoy certain preferential policies in terms of policy support.

### Analysis of legal regulations and policy influencing factors

During the period 2017–2021, several laws, regulations and policy documents have been introduced in China related to environmental protection in the paper industry. The following are some of the important laws, regulations and policies that have prompted companies to pay more attention to water environmental issues and take more effective management measures, thus driving up indicators. In 2015, the State Council promulgated the Water Pollution Prevention and Control Action Plan and the Ten Articles of Water, which provide for the principles, responsibilities, legal liabilities and supervision of water environmental protection; in 2017, the Water Pollution Prevention and Control Law was revised to Prevention and control was regulated, for the paper industry wastewater discharge standards, sewage permit system, water environmental monitoring and assessment and other aspects of the corresponding requirements; 2019 the National Development and Reform Commission, the Ministry of Water Resources jointly issued a "National Water Conservation Action Plan" for industrial enterprises to put forward "water conservation and emission reduction" and "Science and technology innovation" and other closely related, paper enterprises from the technical adjustment of the paper industry industrial transformation, increased investment in environmental protection and water-saving equipment, the construction of a new model of diversification of enterprise water-saving services, so as to achieve the transformation of the results of green water-saving technology as soon as possible. The government from the policy and regulations to further strengthen the protection of water sources and limit the discharge of polluted water sources, for enterprise water resources management and water information disclosure to provide planning and guidance. The two go hand in hand to promote the paper industry intensive water conservation, to the green, low-carbon direction, in the 2020 implementation of the "2020 in accordance with the law to promote backward production capacity exit work program" policy documents to the paper industry and other heavy polluting enterprises were strictly in accordance with the law to eliminate backward products, so that overcapacity contradictions are eased, reducing the discharge of industrial wastewater in the production process, indicating that the paper industry has always Adhere to the policy guidance of green and sustainable development, and actively improve and upgrade water-saving technology to reduce production costs and sewage discharge. In the same year, the Law of the People’s Republic of China on the Prevention of Environmental Pollution by Solid Waste was introduced, which clearly stipulates that China will gradually achieve zero solid waste imports. The continuous adjustment and retrenchment of the waste paper import policy has led to the concentration of enterprises’ foreign waste quotas in large enterprises, the decline of foreign waste ratios year by year, and the rising cost of raw materials. In addition, China has issued a series of policy documents on environmental protection and environmental impact assessment of construction projects, such as the China Agenda for Sustainable Development 2030 and the Law of the People’s Republic of China on Environmental Impact Assessment, which provide guidance and measures to regulate the procedures and contents of environmental protection and environmental impact assessment. Therefore, under the guidance and promotion of national laws and regulations and policy documents, we should strengthen the regulation and restraint of enterprise water pollutant discharge and encourage enterprises to voluntarily disclose water information in order to realize the optimal allocation and intensive, economical and safe use of enterprise water resources.

## Conclusions

In order to better evaluate the level of enterprise water information disclosure quality, this paper constructs a water information disclosure quality index system for paper and paper products enterprises, which improves and supplements the previous evaluation indexes. The projection tracing model is used to analyze A-share paper and product enterprises. Meanwhile, in order to overcome the shortcomings of the traditional model of projection tracing, an improved model based on accelerated genetic algorithm is proposed, which improves the accuracy and robustness of the model. Finally, the effectiveness and adaptability of the method is demonstrated through a case study of water disclosure quality evaluation of 27 paper companies in China, which provides ideas for the study of corporate water disclosure quality evaluation. Our study shows that (1) corporate water disclosure quality indicators include four basic criteria: "relevance", "reliability", "validity" and "comparability", as well as the selection of water recycling efficiency, corporate COD emissions, environmental information exchange with stakeholders, corporate daily water management system plans, goals and strategies, and corporate water management performance evaluation programs as key indicators for evaluation. (2) Introducing accelerated genetic algorithm in the traditional projection tracing model to construct the best projection weights and evaluation values to more accurately and reliably analyze the dynamic evolutionary trend changes of information disclosure related to water resources in paper and paper product enterprises. (3) Analysis of the vertical trend changes of weights and evaluation values from 2017 to 2021 reveals that the disclosure of information about water resources in paper and paper products enterprises shows fluctuations and increases in 2017, but after 2019, enterprises disclose information about water resources tends to slow down, and in the disclosure carrier, relevant reports gradually compress the details of information disclosure about water, which is related to relevant environmental policy changes and enterprise disclosure awareness Weakness is related. And from the tax policy, from technological innovation and application factors, industrial structure transformation, environmental investment governance and social public participation in five categories of influencing factors, a detailed analysis of the trend of change in paper enterprises, (4) from the 2021 weight and rating value of the horizontal trend change analysis, in the rating level, the number of excellent enterprises is less, indicating that enterprises are not high enthusiasm for voluntary disclosure of water resources information, enterprise water information The overall quality of disclosure quality results are less satisfactory; the nature of enterprises, state-owned enterprises slightly better than non-state enterprises, mainly because of the stronger policy sensitivity of state-owned enterprises and its image and reputation of the direct impact on the image of the country; from a regional perspective, Zhejiang Province and Shandong Province in the overall enterprise water information quality evaluation system performance is better, its advantages in raw materials, water resources, geographic location, market demand, technology and innovation and environmental policy rules, while Shanghai and Shaanxi Province evaluation value is at a lower level, mainly by the geographical environment to limit access to water resources, as well as enterprises involved in commercially sensitive information or privacy issues, reluctance to actively disclose water-related information factors. It is worth noting that although the projection tracing imitation with the introduction of genetic algorithm overcomes the subjective factors of the evaluation method to a certain extent, the subjectivity brought by the content analysis method still cannot be avoided. This paper tries to establish a scientific, complete and effective evaluation index system, which will help to achieve the comprehensiveness of the evaluation index and make the definition of the evaluation results clearer and more accurate through the continuous in-depth research conducted by future scholars and thus improve the objectivity of the evaluation results.

We note that these results have some implications for understanding corporate water information disclosure, first, to raise awareness and improve the construction of the legal and regulatory system. In recent years, a number of laws and regulations have been promulgated in environmental protection, but. However, the legal system for environmental information disclosure, especially water information disclosure, is still under development. Specific measurement indicators regarding water information disclosure need to be continuously improved, and specific requirements and content forms for water information disclosure should be introduced as soon as possible. Enterprises in the water information disclosure, daily need to measure, and collect data and timely collation of information. The process will increase the cost of certain fees, due to the lack of mandatory disclosure of enterprise water information, resulting in the disclosure of enthusiasm is not high. The government should lead enterprises to carry out water-saving technology upgrades, promote enterprise green technology innovation, and reduce enterprise water and environmental risks. At the same time, the government’s specific system continues to adapt to changes in the implementation of the enterprise to the reality, to give enterprises a certain amount of financial assistance, so that the ecologically sustainable development of enterprises have a material basis for the protection of positive water information disclosure of enterprises, through green financial means to promote the construction of enterprise water information disclosure system, to mobilize the initiative of enterprises.

In addition, there is a need to improve the system, and the ability to play the main corporate governance. Enterprises are the main body of water information disclosure, in the pursuit of profit maximization at the same time, shoulders social responsibility, high water-sensitive industries should be a more spontaneous initiative to disclose water information, but from the report, only three enterprises independently disclose information related to water information in the annual environmental report, one enterprise independently discloses the annual sustainability report, there are enterprises did not disclose social responsibility report. From the disclosure of information, to the disclosure of formal content, the quality varies, only the disclosure of better indicator data, so that stakeholders have one-sided cognitive enterprise water environment status. Enterprises combine their own reality and the requirements of relevant departments to establish an internal control system for environmental management, responsible for information collection, collation, review and release, to ensure the authenticity and reliability of data disclosure, the main person in charge of the enterprise to enhance the sense of responsibility, the enterprise water resources information disclosure as an important work well arranged and deployed, the enterprise management of water resources information disclosure with business work with the same arrangements, with the implementation, and finally detailed to each The management of the enterprise will arrange and implement the disclosure of water resources information together with the business work, and finally, it will be detailed to each staff position, encourage the staff to actively participate in the activities of information disclosure, and give job promotion and material rewards to the staff who are complete and comprehensive in the information disclosure.

## Data Availability

Upon publication of the paper, the datasets used and analyzed during the study are available from the corresponding author upon reasonable request.
